# Calvarial Metastasis as the Initial Presentation in Hepatocellular Carcinoma: A Rare Entity

**DOI:** 10.1007/s13193-025-02324-6

**Published:** 2025-05-19

**Authors:** Katyayani Kumari, Abdeali Saif Arif Kaderi, Shraddha Patkar

**Affiliations:** GI and HPB services, Tata Memorial Hospital, Homi Bhabha National Institute, Mumbai, India

**Keywords:** Hepatocellular carcinoma, Calvarial metastasis, Swelling

## Abstract

Hepatocellular carcinoma (HCC) presenting as calvarial metastasis (CM) is a rare situation with limited reports in literature. We present a case of a 64-year-old gentleman, who presented with an 8-month history of swelling on the right forehead, associated with decreased vision and size of the right eye. Magnetic resonance imaging revealed a partially hemorrhagic lesion in the right frontal bone, extending into the scalp and causing a midline shift, with intra-orbital and intraconal extension. Positron emission tomography showed multiple bi-lobar liver metastases. The patient was diagnosed with hepatitis B–associated hepatocellular carcinoma on biopsy and was started on dexamethasone, anti-epileptic prophylaxis, and Lenvatinib. He also received focal radiotherapy for the CM. Advanced imaging techniques were crucial in diagnosing the disease’s extent. The patient’s management, involving pharmacological therapy, radiotherapy, and supportive care, highlights the need for a multidisciplinary approach in treating advanced malignancies.

## Introduction

Hepatocellular carcinoma (HCC) accounts for two-thirds of liver malignancies and fourth highest number of cancer-related deaths. Extrahepatic metastasis in HCC at presentation is uncommon (14 to 36.7%) [1] Skeletal metastases are third most common site of metastasis after lung and lymph node, with an incidence of 16.1 to 38.5%. Most common affected bones are vertebrae, pelvis, ribs, skull, humerus, and sternum [2].

We describe a rare presentation of HCC with calvarial metastasis (CM) as a forehead swelling. The incidence of CM in HCC is low (0.5–1.6%), and it is speculated to spread via the Batson’s plexus. In a Japanese autopsy study, only 17 skull metastases were seen (6.1%) among 278 cases of bone involvement. CM as a presenting symptom of HCC is rare, 38 such cases have been reported till date of which 14 cases had solitary CM [3, 4]. CM are found commonly in cancers of breast, follicular and papillary thyroid cancers, lung, prostate, melanoma, Ewing’s sarcoma, and hematological conditions like multiple myeloma, plasmacytoma and cutaneous T cell lymphomas, and rarely endometrial cancer and cholangiocarcinomas [2, 5].

The local treatment option ranges from surgical resection for solitary lesions with neurological deficit, radiotherapy for symptomatic lesions and bleeding ulcers, gamma knife, and ablative therapies [3]. Targeted therapy and immunotherapy such as sorafenib, lenvatinib, atezolizumab and bevacizumab, atezolizumab and cabozantinib, durvalumab and tremelimumab, and pembrolizumab have been reported to improve median survival from 6 to 11 weeks even in bone metastases [4, 6, 7].

## Case Report

**Fig. 1:**
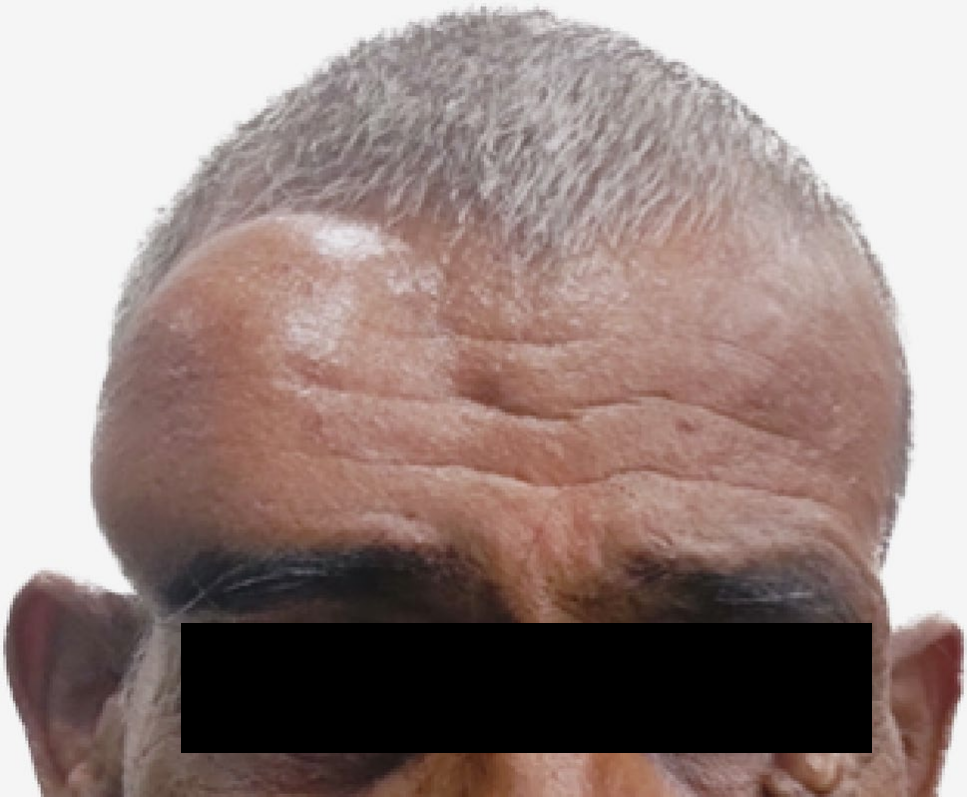
A 64-year gentleman with right forehead firm well-defined swelling of 8 × 8 cm with enophthalmos of the right eye with complaints of diminution of vision in the right eye

**Fig. 2:**
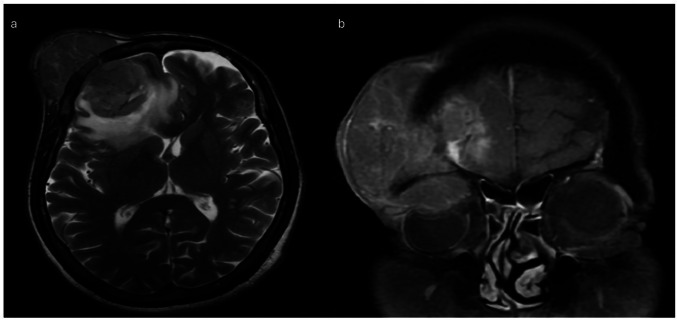
**a** Magnetic resonance imaging with T2 sequence depicting right frontal heterogeneously hyperintense partially hemorrhagic lesion arising from the right frontal bone with associated soft tissue component and intracranial extradural extension. Intraorbital component with mass effect compressing the right frontal lobe with moderate perilesional edema and midline shift is also noted. **b** MRI head T2 coronal view depicting frontal bone lytic lesion with intraorbital and intraconal extension. It shows loss of fat planes with the superior rectus muscle and is abutting the superior aspect of the globe. Other features seen include bone lysis, loss of bone marrow signal, and asymmetry in diploic space

A 64-year gentleman presented to the outpatient department with complaints of swelling on the right forehead since the last 8 months associated with diminution of vision and decrease in size of the right eye from last 3 months. A firm, non-tender, fixed 8 × 8 cm swelling was noted over right forehead, extending up to right eyebrow and stretching the overlying skin. There was enophthalmos of the right eye (Fig. 1). The frontal location of the tumor seemed to cause a pressure on the globe in the anteroposterior direction, resulting in globe compression and enophthalmos. A magnetic resonance imaging (MRI) of the brain and head region revealed a 5.7 × 5.2 × 5.7-cm T1 isointense and T2 heterogeneously hyperintense partially hemorrhagic lesion arising from the right frontal bone with soft tissue component extending into the scalp. Intracranial extradural extension along the right frontal region was noted compressing the right frontal lobe with moderate perilesional edema and a midline shift of 1.1 cm (Fig. 2). Intraorbital and intraconal extension was also present with superior rectus muscle involvement along with abutment of the superior aspect of the globe (Fig. 2). Fludeoxyglucose-^18^ (FDG) positron emission tomography (PET) scan showed an FDG avid CM with 4 cm lesion in the segment VIII of the liver (SUVmax 4.5) with multiple bi-lobar liver metastases. Biopsy from the CM revealed metastatic carcinoma with hepatocytic differentiation. Tumor cells were positive for HepPar I and Glypican (focal) and negative for CK7. On further workup, he was detected to be positive for hepatitis B surface antigen. Alpha-feto protein was 774 ng/mL. PIVKA-II (protein induced in vitamin K absence was 714.42 mAU/mL). He was started on dexamethasone and anti-epileptic prophylaxis. Lenvatinib 8 mg was started as immunotherapy could not be offered due to cost constraints. Focal radiotherapy (30 Gy in 10 fractions) was given to the CM.

CM can be one of the rare initial presentations of HCC. While the prognosis is dismal, a biopsy with immunochemistry is the key to diagnosis and early palliation with systemic therapy is the way forward.

## Data Availability

The data that support the findings of this study are available from the corresponding author upon reasonable request.

## Declarations

The corresponding author is section editor for the journal.
